# Retracted: The Studies of Chlorogenic Acid Antitumor Mechanism by Gene Chip Detection: The Immune Pathway Gene Expression

**DOI:** 10.1155/2020/1237865

**Published:** 2020-12-21

**Authors:** Journal of Analytical Methods in Chemistry

*Journal of Analytical Methods in Chemistry* has retracted the article titled “The Studies of Chlorogenic Acid Antitumor Mechanism by Gene Chip Detection: The Immune Pathway Gene Exdmpression” [1]. Confidential information from Sichuan Jiuzhang Biological Science & Technology Co., Ltd. (previously known as Sichuan Jiuzhang Biochemical Engineering Science and Technology Development Co., Ltd.) was published without permission, as confirmed by a court judgment. A previous corrigendum that acknowledged the involvement of the company was not sufficient [2].
